# A Tool for the Assessment of Swallowing Safety and Efficiency in Adults: Turkish Adaptation of Boston Residue and Clearance Scale

**DOI:** 10.1007/s00455-024-10706-1

**Published:** 2024-05-04

**Authors:** Samet Tosun, Saime Seyhun Topbaş, Elif Aksoy

**Affiliations:** 1 Faculty of Health Sciences, Department of Speech and Language Therapy, Biruni University, İstanbul, Turkey; 2 School of Health Sciences, Department of Speech and Language Therapy, İstanbul Medipol University, İstanbul, Turkey; 3 Tesvikiye ENT Group, İstanbul, Turkey

**Keywords:** Dysphagia, Validity and reliability, Residue, FEES

## Abstract

The objective of this study was to create a Turkish language adaptation of the Boston Residue and Clearance Scale (BRACS), a validated and reliable tool. The BRACS scale was first translated into Turkish and a Turkish version was subsequently developed. Fiberoptic endoscopic examination of swallowing (FEES) was administered to collect data from 25 dysphagic patients who were hospitalized after a stroke. The recorded films were subjected to editing procedures to ensure their appropriateness for the assessment of swallowing disorders and were then dispatched to a panel of five speech and language therapists for evaluation using the adaptation of the BRACS instrument. The scoring by the experts was evaluated using both explanatory factor analysis (EFA) and confirmatory factor analysis (CFA). Convergent validity, item reliability, and construct (composite) reliability were measured by calculating the average variance extracted (AVE) values. For the 12 location items, EFA revealed 3 main latent factors: the laryngeal vestibule and the oropharynx and hypopharynx. The Turkish BRACS had excellent inter-rater reliability (Krippendorff’s alpha coefficient values ranged from 0.93 to 0.95) and high internal consistency (Cronbach’s alpha values ranged from 0.88 to 0.93). Inter-rater ICCs for the first and second sessions were 0.83 and 0.85, respectively. CFA showed that all fitted criteria reached acceptable or perfect fit levels. The findings indicated that the proposed factor structure was validated. The AVE values are between 0.61 and 0.73 which was taken as evidence of convergent validity. The Turkish adaptation of the BRACS tool demonstrates both reliablity and validity, rendering it a useful and credible tool for assessing residual severity, particularly in clinical settings.

## Introduction

The human capacity to swallow, which naturally begins with the oral pathway, is a process orchestrated by the harmonious interaction of anatomical and physiological systems located in the oropharyngeal and esophageal regions. This mechanism operates in a sophisticated manner. Swallowing function could be disrupted by dysphagia, a multifaceted condition arising from various etiological factors that consequently present individuals with nutritional challenges. Stroke is another prevalent medical condition often associated with dysphagia, particularly in cases with neurological origins [1].

Various stroke factors, including the size and location of the stroke lesion, have significant implications such as dysphagia, and the degree of its severity. The prevalence of dysphagia following a stroke has been documented to be as high as 78%, with researchers reporting the occurrence of aspiration pneumonia in approximately 48% of affected individuals [2]. These deteriorations have a cumulative impact on patients’ lives, negatively affecting them and necessitating higher healthcare expenses [3]. Consequently, immediate identification and timely diagnosis of dysphagia can lead to prompt resolution of the symptoms and consequences associated with the impairment of swallowing function [4]. The diagnosis of a swallowing issue can be readily accomplished using two distinct instrumented imaging techniques, which are widely regarded as the gold standards in the field: : the modified barium swallow study (MBSS) and the fiberoptic endoscopic examination of swallowing (FEES). Both of these swallowing tests offer health professionals an unbiased assessment. If there is a failure in the occurrence of a swallowing, or if there is an abnormal sequence, timing, or intensity of these events, it can lead to reduced swallowing safety and efficiency. These two tests are used to understand whether the patient safely and efficiently swallow. The existing literature indicates that both of these fundamental evaluation methodologies are capable of providing objective instrumental assessments of swallowing difficulties associated with residues retained in the oral and pharyngeal pathways.

A scientifically relevant residue scale for determining residue retention must evaluate three crucial elements: the quantity of the residue, the location of the residue, and the patient’s reaction to the residue. First, an increase in the quantity of residue has been found to exacerbate the physiological decline in swallowing function. Second, the location of the residue plays a crucial role in determining the likelihood of aspiration. Third, the patient’s lowered capacity for spontaneous swallowing against the residue, as well as their diminished sensation and awareness of the presence of residue, further contribute to an increased risk of aspiration [5]. The MBSS has had numerous residual score scales developed specifically for its use [6, 7], including a subjective assessment measures commonly employed in clinical settings to evaluate the residual rate through the utilization of binary questions (indicating presence or absence) [8], ordinal ratings (ranging from absent to heavy) [9, 10], or the estimated ratio of the bolus upon swallowing [11, 12]. However, these subjective assessments raise significant concerns due to their low inter-rater reliability. By contrast FEES provides greater sensitivity than MSS for detecting pharyngeal residues [13]. Notably, neither method assesses the efficacy of residue clearance, also referred to as residue management, in terms of quantity and distribution [5, 13, 14].

Thus far, only one study has been published in the Turkish language on the assessment of the severity, validity, and reliability of pharyngeal residual measurements [15], and that study did not include the aspect of residual management. Contrary to other tools, BRACS provides advantages in accurately determining the location and quantity of residue on both the oropharynx and hypopharynx for FEES when patients with oropharangeal dysphagia are examined. The aim of the present work was therefore to perform a validity and reliability study of the Boston Residue And Clearance Scale (BRACS) tool after its adaptation for the Turkish language. The BRACS tool, developed by researchers [16] was designed to evaluate the effectiveness of residue clearance in patients and encompasses, several key aspects including the location, quantity of the residue as well as the efficacy of patients’ ability to eliminate the residue in a clinically useful manner. By using BRACS, patient’s response to residue could be assessed. Given the scarcity of comparable studies in the domain of swallowing difficulties, this investigation is anticipated to contribute substantial scientific information. The aim of this study was to address the following research questions:

1. Is the developed Turkish language adaptation of the Boston Residue and Clearance Scale a valid instrument scale?
2. Is the developed Turkish language adaptation of the Boston Residue and Clearance Scale a reliable instrument scale?

## Materials and Methods

### Data Collection Protocol

**Fig. 1:**
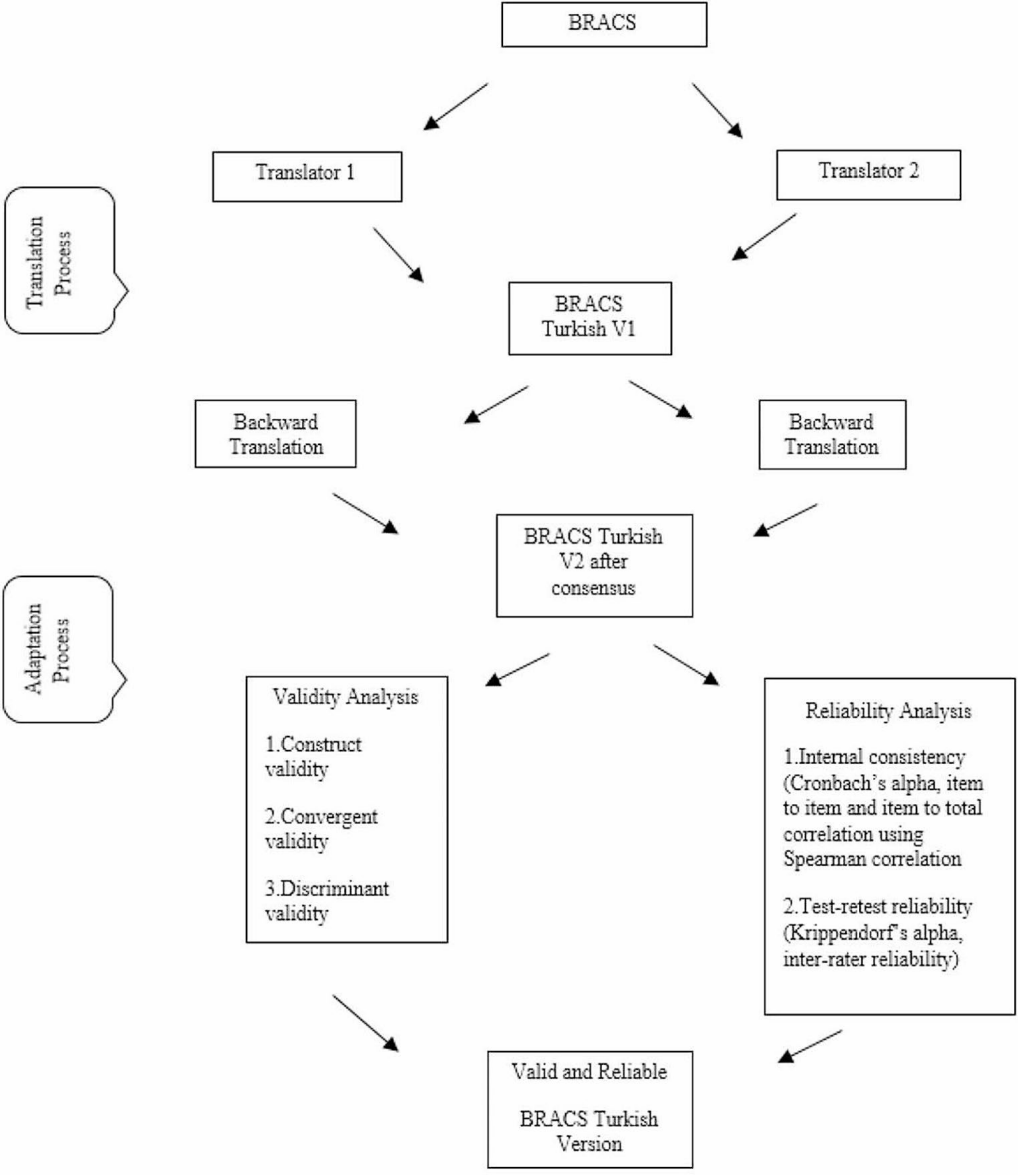
Research methodology flow chart

The data related to the adaptation of BRACS in Turkish were obtained FEES on a sample of patients who were receiving treatment for swallowing difficulties at Liv İstinye University Hospital and Bağcılar Medipol Mega University Hospital. The study involved administering food to the stroke patients in a total of four different consistencies: thin liquid, yoghurt, puree, and solid consistency; all foods were artificially colored with green food dye. When patients were given these boluses, the application of FEES was administered by the researchers and the captured videos were saved. They were then digitally converted to electronic media for assessment by the raters. The researchers organized the videos in a suitable manner to facilitate scoring by the raters. A total of 102 swallowing videos obtained from FEES videos were assigned numerical identifiers using Davinci Resolve 18 software and organized for the purpose of scoring. All 102 separate videos were randomly numbered and merged into a single film by using Davinci Resolve 18 software to make rating easier. After having created this single film, researchers created another randomly numbered second film to send it to raters when they completed the first compliation. Second film was given to raters after two weeks when the raters completed their first asessment of the videos. The audio in the videos was intentionally muted to prevent raters from being misled or influenced by auditory cues. A total of 102 edited videos and the Turkish adaptation of BRACS were delivered to the raters. Upon viewing of the videos, the raters assigned scores to the 12-item scale ranging from 0 to 3 points (0 = none, 1 = mild, 2 = moderate, 3 = severe) based on the extent of residue observed in the video footage reviewed by the raters. Each rater scored a total of 102 videos. The research was conducted as shown in the flow chart in Fig. 1.

The components on BRACS are used to determine the residue presence and quantity on Pharyngeal wall, Base of tongue, Vallecular, Epiglottic tip, Lateral channel, Piriform sinus, post-cricoid, Arytenoid, Aryepiglottic, Inter-arytenoid, Laryngeal surface of the epiglottis, Laryngeal surface of the aryepiglottic fold, Ventricular folds, Anterior commissure, Vocal fold, Posterior commissure [16]. The Turkish adaption of the English version of BRACS [16] was undertaken by a multidisciplinary team consisting of an otolaryngologist, three speech and language therapists (SLTs), and one statistician. Forward translation from English to Turkish was carried out by two independent SLTs who were experienced in swallowing disorders. The backward translation was implemented by two independent SLTs who were blinded to the original English version. Each member of the team was a native Turkish speaker. The research team conducted a thorough analysis of misunderstandings and subsequently made improvements to the translated Turkish BRACS, resulting in its current version.

### Patient Group

The study involved the assessment of 12 male and 13 females patients (48% male; 52% female). Demographic characteristics of patient group are presented in Table 1. They were aged between 40.5 and 80.9 years. Patients were receiving treatment for post-cerebrovascular accident and experiencing dysphagia at Liv İstinye University Hospital and Bağcılar Medipol Mega University Hospital. FEES was administered to each of them.

**Table 1:** Demographic characteristics of patient population

Patient Population	Diagnosis (N)	Gender N (%)		Age (years) Mean ± SD (min-max)
25 patients with oropharyngeal dysphagia	Cerebrovascular Accident (25)	M 12 (48%)	F 13 (52%)	67.5 ± 11.7 (40.5–80.9)

### Characteristics of Evaluators

Five speech and language therapists (SLTs) with 9.40 ± 2.30 years of experience were included as participants in the study to assess the videos of the patients with dysphagia for the validity and reliability of the Turkish adaptation of the BRACS. They participated this study as raters. 102 FEES videos obtained from the patients were given to raters. By using Turkish version of BRACS, they rated the FEES videos. No information was provided to raters about patients and BRACS. Audio in the videos was off to avoid any influence or cue. Raters were not given a time limit to assess the videos. They used as much time as they needed to complete the rating process.

### Statistical Analyses

The data gathered in the study were analyzed using the LISREL 8.7 and SPSS 25.0 software packages. In this study, the researchers utilized the SPSS 25.0 software package to conduct an explanatory factor analysis (EFA) and to determine the internal consistency factor. The LISREL 8.7 software was also employed for confirmatory factor analysis (CFA). The significance levels for the analyses were accepted as *p* = 0.01 and *p* = 0.005, respectively.

The researchers assessed the validity of the scale by conducting EFA and CFA consecutively. Multiple fit indices were employed to assess the adequacy of the model in CFA. This research considered the Chi-square goodness of fit index, along with other fit criteria, such as the incremental fit index (IFI), comparative fit index (CFI), root mean square error of approximation (RMSEA), goodness of fit index (GFI), and root mean square residual (RMR) [17]. The characteristics of the cases and raters were analyzed by assessing frequency and percentage distributions as well as by calculating mean and standard deviation values. A varimax axis rotation was also conducted. The validity of the structure model derived from the EFA, in conjunction with the CFA, was assessed.

The reliability of the scale was assessed using Cronbach’s alpha, which measures the internal consistency of the items by retesting. The research employed the internal consistency approach to calculate the reliability levels of the scales. The reliability criteria of Cronbach’s alpha and Krippendorff’s alpha were utilized for this purpose [18]. Subsequently, the item-total correlation was computed, and an analysis was conducted to assess the impact of removal of any scale item on the Cronbach’s alpha values.

## Results

### Explanatory Factor Analysis

**Fig. 2:**
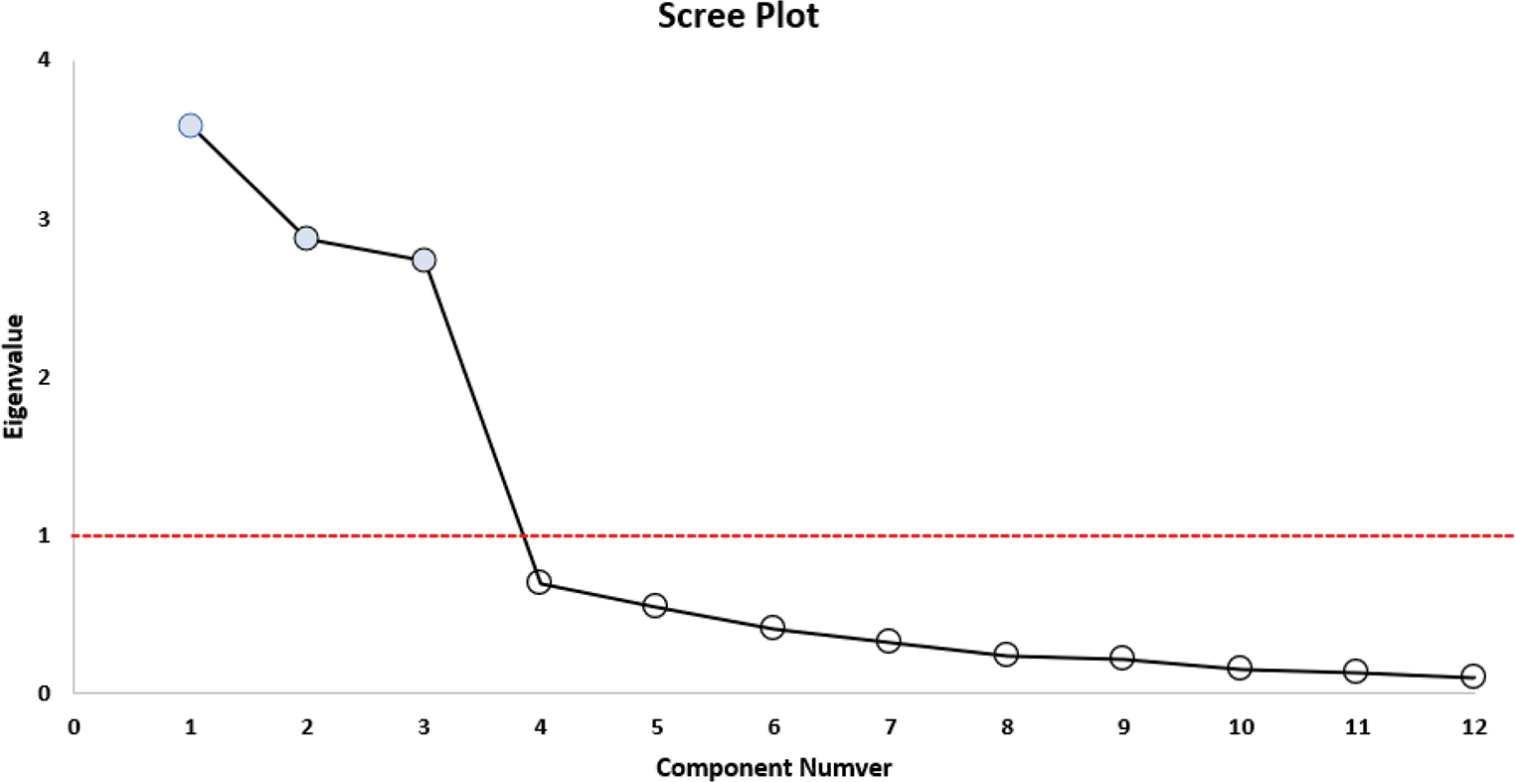
Scree plot of the scale

Initially, the appropriateness of the sample was assessed on the 12-item BRACS to determine its suitability for factor analysis. The Bartlett’s test of sphericity yielded a chi-square value of 7892.949 (*p* < 0.001), indicating a significant departure from sphericity. The Kaiser–Meyer–Olkin measure of sampling adequacy also yielded a value of 0.861, suggesting that the sample size was sufficient for conducting the analysis. Three components were derived from the BRACS using a basic scree-plot test and the eigenvalue > 1.0 criterion, as depicted in Fig. 2 which displays the scree plot to visually represent the eigenvalue.

The expected values for the correlation coefficients for the relationships between the items in the scale and the other items should not be less than 0.30 [19]. As shown in Table 2, the correlation coefficients were all equal to or greater than 0.30. Based on this criterion, there was no need to exclude any item from the scale. The variation of the three factors, which underwent rotation using the varimax approach, accounted for 76.60% of the total variance (Table 1). Factor 1, consisting of six measures, explained 29.88% of the variance and was identified as the Laryngeal Vestibule. Factor 2, consisting of three components, was shown to explain 23.94% of the variation and was identified as the Hypoharynx. Factor 3 (three items) accounted for 22.78% of the variance and was designated the oropharynx. Collectively, 76.60% of total variance was accounted for by these three factors. Inter-arytenoid space and Left arytenoid & Left aryepiglottic fold did not show high loading on any factors. The assessment of the reliability of the BRACS dimensions involved the utilization of Cronbach’s alpha coefficient and the examination of item-total correlations within each dimension. In this particular scenario, the criterion for acceptability was a minimum value of 0.70 for the Cronbach’s alpha coefficients [20, 21].

**Table 2:** Explanatory factor analysis and item analysis results

Items	Factors			Item scale relation
	1	2	3	
1.Lateral pharyngeal wall, Posterior pharyngeal wall			0.813	0.677
2.Base of tongue			0.876	0.616
3.Valleculae, Tip of epiglottis			0.837	0.634
4.Left lateral channel & Left piriform sinus		0.738		0.769
5.Right lateral channel & Right piriform sinus		0.683		0.781
6.Post-cricoid area		0.807		0.721
7.Left arytenoid & Left aryepiglottic fold	0.544			0.643
8.Right arytenoid & Right aryepiglottic fold	0.722			0.698
9.Inter-arytenoid space	0.539			0.752
10.Laryngeal surface of epiglottis	0.801			0.660
11. Laryngeal surface (side walls) of aryepiglottic fold & False vocal folds	0.848			0.726
12. Anterior commissure, True vocal folds, Posterior commissure	0.832			0.751
Reliability	0.909	0.888	0.889	0.931
Eigenvalue	3.586	2.873	2.733	
Variance (%)	29.88	23.94	22.78	76.60

The Kaiser–Meyer–Olkin (KMO): 0.861; Bartlett’s Test of Sphericity = X2(66) = 7892.949; *p* = 0.001

### Confirmative Factor Analysis

The CFA was conducted to evaluate the fit of the model. Using LISREL 8.7, and the model parameters was estimated using the maximum likelihood method. LISREL 8.7 offers a comprehensive set of goodness-of-fit measurements. The use of model fit metrics in CFA can be categorized into three types: absolute fit, incremental fit, and parsimonious fit. This study employed several statistical measures including the chi-square test, which is a minimum fit function test; ; the RMSEA; the GFI; and the SRMR. The incremental fit measures included in this study were the adjusted goodness-of-fit index (AGFI), normed fit index (NFI), Tucker–Lewis index (TLI), and CFI. For the scale to be considered acceptable, the derived goodness-of-fit criteria must be within the predetermined minimum acceptable limits. The primary investigation of the proposed scale revealed a decrease in the fit criteria, which fell within the acceptable and optimal fit range. Examination of the fit criteria values derived from the CFA revealed that the ratio of the chi-square value to the df value was 4.219, which was an acceptable fit level. The RMSEA value was 0.079, which is also an acceptable fit level. All the other fit criteria also indicated acceptable or perfect fit levels. Given these findings, the proposed factor structure was deemed to have been validated (Table 2).

Because all 12 clinical items had an item-total correlation value exceeding 0.30, they were concluded that to have an adequate level of measuring power. As shown in Table 3, the correlations between the scale items and the average score acquired from the scale varied from 0.684 to 0.822 and showed statistical significance at a level of *p* < 0.01. These findings indicated the absence of any issues regarding the consistency of the items with each other. Each of these items could be used in BRACS.

**Table 3:** Scale’s fit measures

X^2^/df	RMSEA	CFI	GFI	AGFI	NNFI	NFI	RMR	SRMR
4.219	0.079	0.97	0.91	0.90	0.97	0.97	0.053	0.030

**Fig. 3:**
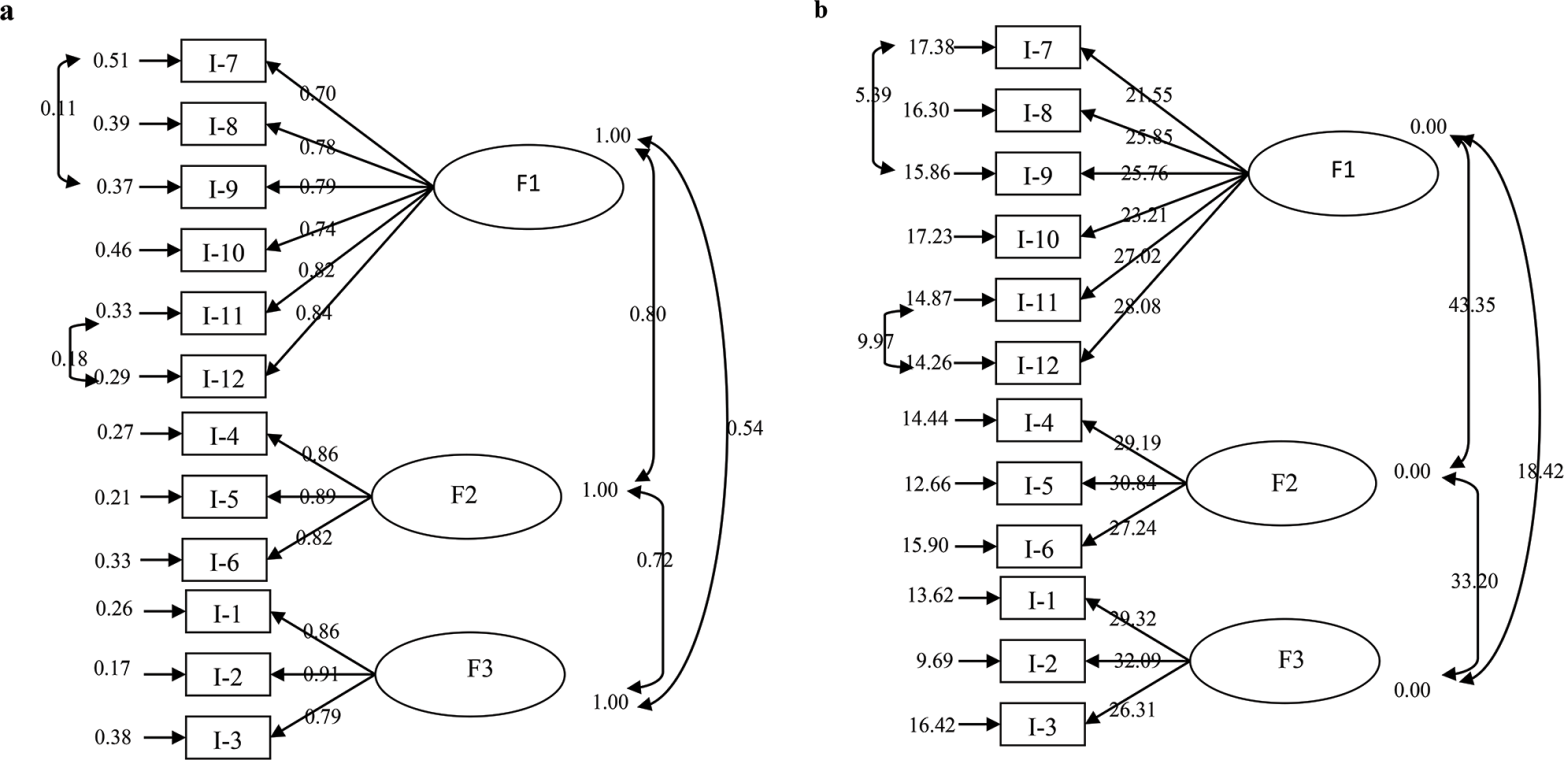
**a** Scale’s CFA Result *Chi-Square = 206.76, df = 49, p-value = 0.00001, RMSEA = 0.079*, **b** T Values of the Scale’s*Chi-Square = 206.76, df = 49, p-value = 0.00001, RMSEA = 0.079*

Figure 3a and b show the standardized beta coefficients obtained from the CFA conducted using the LISREL 8.7 software to assess the validity of the proposed factor structure. Figure 3a clearly shows that revisions were required between items 7–9 and 11–12 based on the CFA results of the scale. This adjustment was necessary because the compliance criteria were somewhat above the acceptable level during the initial phase. The t-values corresponding to the scale factor loads are given in Fig. 3b.

The acceptability of the factor loadings for the items on the scale was confirmed by CFA, resulting in values ranging from 0.70 to 0.91. The t-values, which indicate the statistical significance of the relationships between the items and latent variables, were significant at the *p* < 0.01 level. Additionally, all values exceeded the threshold of 2.58 ( Table 3). Compliance criteria were taken into consideration while determining the acceptability of the CFA model. Table 4 shows the measures acquired from CFA. All measures had acceptable and optimal fit requirements as previously outlined [17].

**Table 4:** Scale items and scale total correlation values

Item No	r	p
1.Lateral pharyngeal wall, Posterior pharyngeal wall	0.738	0.001**
2.Base of tongue	0.684	0.001**
3.Valleculae, Tip of epiglottis	0.700	0.001**
4.Left lateral channel & Left piriform sinüs	0.817	0.001**
5.Right lateral channel & Right piriform sinüs	0.822	0.001**
6.Post-cricoid area	0.775	0.001**
9.Inter-arytenoid space	0.706	0.001**
10.Laryngeal surface of epiglottis	0.747	0.001**
11. Laryngeal surface (side walls) of aryepiglottic fold & False vocal folds	0.799	0.001**
12. Anterior commissure, True vocal folds, Posterior commissure	0.714	0.001**

**p < 0.01

For a scale to be considered acceptable, the derived goodness of fit criteria must be within the predetermined minimum acceptable limits. The primary investigation of the proposed scale revealed a decrease in the fit criteria, which fell within the acceptable and optimal fit range. All the other fit criteria indicated acceptable or perfect fit levels. Based on these data, the proposed factor structure was deemed to have been validated.

### Convergent Validity

The calculation of convergent validity, item reliability, and construct (composite) reliability involved the computation of AVE values, as per the guidelines proposed previously [22]. The concept of item reliability describes the extent to which the variability in an item can be attributed to the underlying construct as opposed to measurement error. Convergent validity is established when an item demonstrates a minimum reliability coefficient of 0.50, a statistically significant t value, or both.

The evaluation of a measurement model often relies on the composite reliability of each construct, which is a key measure. A frequently accepted threshold for satisfactory composite reliability is 0.70 [21]. The computed composite reliability values for each sub-scale in the present study were 0.70 or above (Table 5).

**Table 5:** Scale’s fit measures

X^2^/df	RMSEA	CFI	GFI	AGFI	NNFI	NFI	RMR	SRMR
4.219	0.079	0.97	0.91	0.90	0.97	0.97	0.053	0.030

X^2^: Chi square, Df: Degrees of freedom, RMSEA: Root mean square error of approximation, CFI: Comparative fit index, GFI: Goodness of fit index, AGFI: Adjusted goodness of fit index, NNFI: Non normed fit index, NFI: Normed fit index, RMR: Root mean residual, SRMR: Standardized root mean squared residual

For BRACS scores, inter-rater ICCs for the first and second sessions were 0.83 and 0.85, respectively. The measurement model’s reliability was assessed by examining the CR values as the CR value in Table 5 exceeded the threshold of 0.70. The measurement model’s convergent validity were confirmed, the AVE value exceeded the threshold of 0.50. These values were ascertained to establish the scale’s validity and reliability within the context of the investigation. Taking obtained AVE and CR values under consideration, Turkish version of BRACS is a valid and reliable tool to assess residue presence in patients with dysphagia (Table 6).

**Table 6:** AVE and CR values of scale dimensions

Factors	AVE	CR
Laryngeal vestibule	0.61	0.90
Lower pharynx	0.73	0.89
Upper pharynx	0.73	0.89

**Fig. 4:**
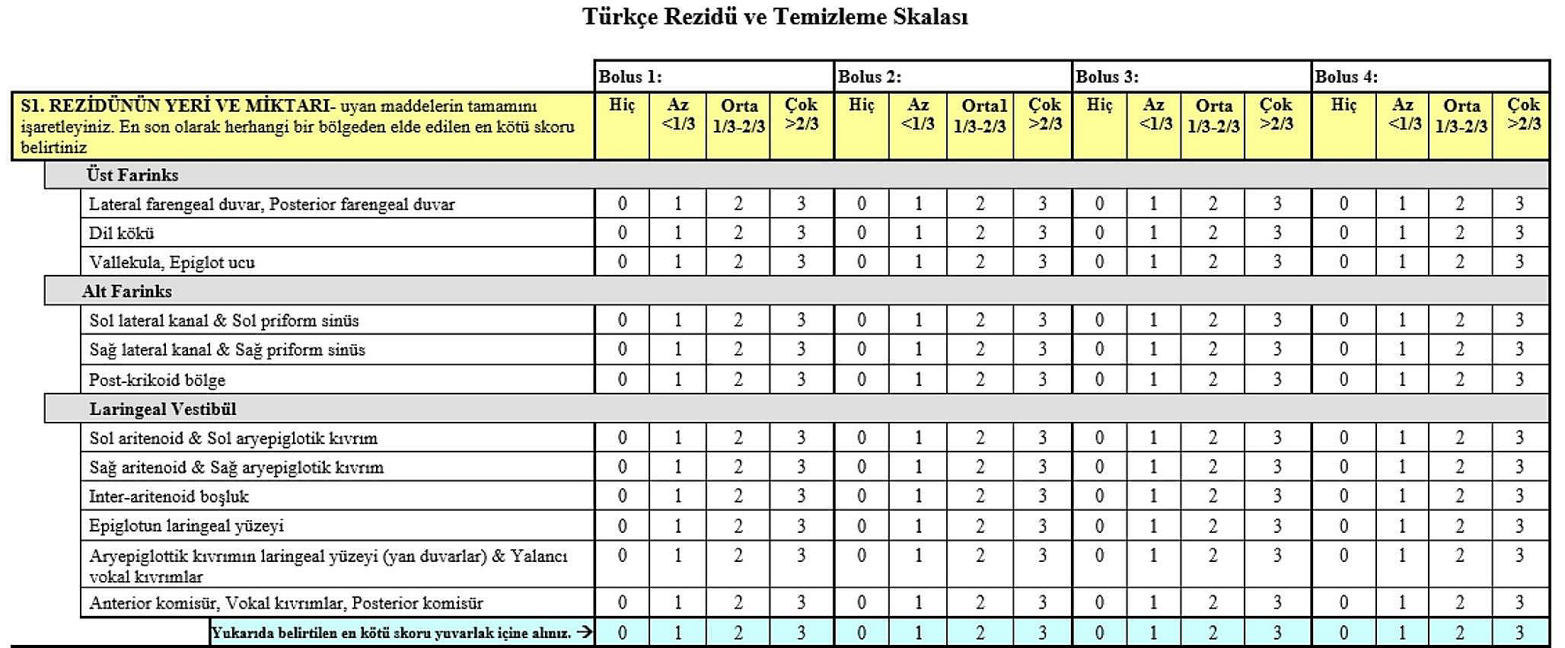
Turkish version of BRACS

Following the completion of the reliability and validity assessments, the final version of the Turkish BRACS was developed, as depicted in Fig. 4.

## Discussion

The aim of this study was to translate, adapt and validate BRACS. The Turkish version of the BRACS was modified with the objective of assessing the severity of residue, and was subsequently examined to determine its concurrent validity and reliability. The construct validity of the Turkish BRACS was assessed using EFA and CFA. The application of EFA indicated no need to eliminate any item from the scale. The variance was accounted for by a three-factor structure consisting of Factor 1, Larengeal Vestibül (Laryngeal Vestibule), Factor 2, named as Alt Farenks (Hypopharynx), and Factor 3, identified as Üst Farenks (oroharynx). These factors collectively explained 76.60% of the variance. Examination of the CFA and the three-factor structure revealed that they exhibited strong fit values.

Although only EFA was used on the items created in the original study, both EFA and CFA were conducted in the BRACS Turkish version. Based on the findings derived from the CFA carried out in this investigation, it was determined that, in conjunction with the EFA results acquired in the initial study, there was no need to eliminate any items from the scale. The findings support the initial investigation. The initial study had inter-rater internal consistency coefficients of 0.81 and 0.80 for the first and second sessions, while our study had inter-rater ICCs of 0.83 and 0.85 for the same sessions (rater 1 = 0.83, rater 2 = 0.85, rater 3 = 0.84, rater 4 = 0.83, rater 5 = 0.85). The data acquired from our investigation demonstrates an acceptable degree of inter-rater reliability, consistent with the findings of the original study.

The results of this study agreed with previous findings reported by researchers [16]. The assessment of the measurement of scale reliability using the internal consistency of items gave Cronbach’s alpha values for both the internal consistency-based factor total scores and general total scores that ranged from 0.88 to 0.93. Furthermore, the generated three-factor structure had satisfactory values for both item reliability and construct reliability. The AVE value was acceptable in all subscales, and the correlation among subdimensions had a moderate level of significance. The AVE value for discriminant validity was satisfactory for all three dimensions.

The findings of this study support the Turkish version of BRACS as a viable and valid measure for assessing the severity of pharyngeal residue. The initial study involving an EFA of the 12 location questions in BRACS identified three primary latent variables, but a CFA was not performed. Conducting a CFA for validity and reliability revealed relationships for all 12 items, which were then confirmed. The anatomical structures were designated as the “laryngeal vestibule,” “hypopharynx,” and “oropharynx.” The Turkish version demonstrated significant levels of inter–rater and test–retest reliability. The high reliability of the training session may be attributed to several factors, including the provision of clear instructions and the absence of time limit imposed on the to score patients. Notably, previous studies [12, 23] that did not explicitly define scoring criteria or offer training sessions were unable to achieve comparable levels of reliability. While it is true that health professionals may need to invest additional time in scoring residue due to the need to comprehend scoring criteria and employ frame-by-frame analysis, these procedures are essential to ensure that the Turkish version of BRACS maintains a high level of reliability. The high internal consistency of the BRACS scale indicates that all the elements included in the scale are strongly interrelated and deemed relevant.

A further point to consider is that the scarcity of Turkish scales [15] that possess both validity and reliability for assessing the severity of swallowing disorders following FEES evaluations renders the task of locating this type of scale a relatively insignificant imposition for physicians, despite the time-consuming nature of performing the tasks. A scoring device of this complexity may prove valuable in the context of research, although it may prove excessively burdensome for regular clinical practice. The factor analysis conducted here identified three characteristics that could serve as a guide for determining residue severity in a clinical setting. By exclusively utilizing the broader designations of “laryngeal vestibule,” “hypopharynx,” and “oropharynx,” our scale achieves a higher level of clarity. This would facilitate its routine application in clinical settings.

This finding also implies that the inclusion of all the components in the scale is necessary for an accurate assessment of the severity of residue. Furthermore, the significance of timely identification of oropharyngeal dysphagia, classification of symptom severity, and assessment of therapeutic outcomes is emphasized in the literature [1, 2, 4]. The implementation of the Turkish version of BRACS is anticipated to provide benefits for clinicians in facilitating their professional duties, while concurrently generating substantial positive impacts on the overall well-being of individuals suffering from swallowing disorders. In summary, upon diagnosis of dysphagia, the therapy process can be initiated immediately. The presence of pharyngeal residue during instrumental swallowing examinations can potentially impact the risk of aspiration, as indicated by clinical judgment [24]. From a therapeutic perspective, the quantity and extent of the aspiration obtained are significant factors that impact the rehabilitation process and clinical results. Individuals who experience aspiration of food and liquids into their airway face an elevated susceptibility to the development of pneumonia [25]. By using Turkish version of BRACS, clinicians can determine whether patients are at risk of penetration and aspiration. BRACS has a high diagnostic and treatment value for improving airway safety and efficiency.

We highlight its applicability as a residue assessment tool in patients with pharyngeal dysphagia or patients with a risk of dysphagia. Turkish version of BRACS can assess patients’ residue location, quantity, severity and management of it. Using of BRACS in the clinical setting could provide clinicians with very valuable information regarding patients’ swallowing safety and efficiency and take the required precautions for pharyngeal dysphagia. Residue management is a must for patients’ overall health.

The present study has a few limitations. One of them is that our study did not include swallowing difficulties of various etiologies although this cannot be considered a strict limitation, as the manifestation of swallowing symptoms demonstrates minimal variation across different etiologies [3, 26]. The fact that only a relatively small number of experts participated in our study is another limitation of this study. Future research studies on larger sample size of evaluators are required to confirm the results of present study. Additionally, it is important to note that incorporation of additional raters who are healthcare professionals engaged in dysphagia therapy but who are not SLTs would likely result in enhanced reliability and consensus validity. Lastly, when this research was initiated no Turkish version of residue scale existed to compare our results from BRACS. Provided that other scales are developed, the results should be compared using the scales. In future studies, our recommendation is that researchers incorporate additional healthcare professionals as raters.

## Conclusion

A novel measurement instrument designed to evaluate the issue of residue observed during a FEES test has been developed for the Turkish dysphagia literature. The Turkish BRACS exhibits acceptable levels of reliability, convergent validity, concurrent validity, and discriminant validity. The scale encompassed items that were suitable for evaluating the severity and location of residue, the patient’s reaction to the residue, and the efficacy of any clearance swallows. In accordance with the recommendations put forward in the original research article, additional modifications were implemented to enhance the clinical applicability and validity of BRACS, building upon the findings from the explanatory and confirmatory factor analysis.

## Data Availability

Data are available through the corresponding authors upon reasonable request.
